# Temperature-Triggered Colloidal Gelation through Well-Defined Grafted Polymeric Surfaces

**DOI:** 10.3390/gels3020021

**Published:** 2017-06-03

**Authors:** Jan Maarten van Doorn, Joris Sprakel, Thomas E. Kodger

**Affiliations:** Physical Chemistry and Soft Matter, Wageningen University & Research, Stippeneng 4, 6708 WE, Wageningen, The Netherlands; janmaarten.vandoorn@wur.nl (J.M.v.D.); joris.sprakel@wur.nl (J.S.)

**Keywords:** colloid gel, grafting density, temperature-triggered gelation, surface initiated atom transfer radical polymerization

## Abstract

Sufficiently strong interparticle attractions can lead to aggregation of a colloidal suspension and, at high enough volume fractions, form a mechanically rigid percolating network known as a colloidal gel. We synthesize a model thermo-responsive colloidal system for systematically studying the effect of surface properties, grafting density and chain length, on the particle dynamics within colloidal gels. After inducing an attraction between particles by heating, aggregates undergo thermal fluctuation which we observe and analyze microscopically; the magnitude of the variance in bond angle is larger for lower grafting densities. Macroscopically, a clear increase of the linear mechanical behavior of the gels on both the grafting density and chain length arises, as measured by rheology, which is inversely proportional to the magnitude of local bond angle fluctuations. This colloidal system will allow for further elucidation of the microscopic origins to the complex macroscopic mechanical behavior of colloidal gels including bending modes within the network.

## 1. Introduction

Colloidal particles are of significant importance to various fields in science and engineering and to consumer products, such as foods and paints. Upon inducing sufficiently strong attraction to a colloidal suspension, colloidal particles will aggregate and form a mechanically rigid percolating network above a critical volume fraction [1]. These structures, known as colloidal gels, can be regarded as a model for soft heterogeneous solids. Differing from polymeric gels, the bonds between particles in colloidal gels have a non-permanent nature enabling bonds to reform and individual particles to rearrange due to mechanical deformation or thermal fluctuations [2,3,4]. These rearrangements mainly govern the mechanical behavior of these soft solids and are of paramount importance to understanding the mechanics of soft heterogeneous solids [5,6].

Many efforts studying the particle dynamics within colloidal gels focus on the attraction strength as a control parameter. Systematic investigations on colloidal gels typically employ a depletion attraction [7], where both the range and depth of interaction may be tuned. However, apart from longitudinal fluctuations such as detaching and attaching, particles can also exhibit transverse modes of rearrangement such as sliding [3]. Where the first mode is mainly influenced by the inter-particle potential, the details of the other modes are difficult to unravel, but thought to be governed by the surface properties of the particles such as their friction coefficients [8]. The implications of such parameters on the assembly of colloidal systems may be profound, and are only briefly discussed in the literature; this is partly due to the fact that there does not yet exist an experimental means to investigate their effects.

In this paper, we synthesize a colloidal model system that is suitable for systematically studying the effect of particle surface properties such as grafting density and chain length, on the dynamics within colloidal gels. We control the grafting density and chain length by using surface initiated atom transfer radical polymerization (ATRP): The grafting density is tuned by copolymerizing a known volume of an ATRP initiator-monomer during particle formation and the chain length is tuned by adding a sacrificial initiator to the bulk solution during the ATRP reaction. We grow a temperature sensitive polymer, poly(*N*-isopropylacrylamide), from the particle surface to alter the interparticle potential dynamically. After inducing an attraction between particles by heating, a clear dependence on the magnitude of local bond angle fluctuations and linear mechanical behavior of the gel arises from both the grafting density and chain length. Lastly, we disperse these particles in a refractive index matching aqueous solution allowing for 3D confocal imaging during gelation.

## 2. Results and Discussion

**Figure 1:**
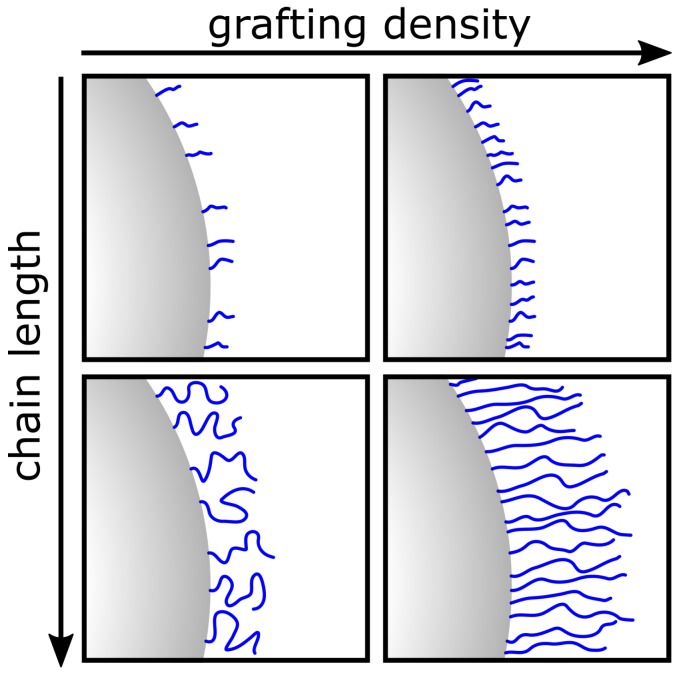
Controlled grafting density and chain length using surface initiated atom transfer radical polymer (ATRP) of poly(*N*-isopropylacrylamide) (pNIPAM).

The origin of the interparticle attraction between colloidal particles can be varied; common examples are depletion [7], electrostatic [9], or van der Waals; however, these sources of attraction cannot be easily triggered. Here, we induce inter-particle attraction using a temperature sensitive surface grafted polymer, poly(*N*-isopropylacrylamide) (pNIPAM) [10]. This polymer has a Lower Critical Solution Temperature (LCST) in water around 32 °C. Above this temperature, the polymers expel water and demixes from the aqueous solution which induces interparticle attraction [11]. When the temperature is lowered below the LCST, the polymer solubility is enhanced, resulting in good solvent for T ≪ LCST, and the interparticle potential becomes sterically repulsive. The precise value of the LCST is sensitive to the composition of the solvent [12] and as a result, we design our system to be stable in water. One of the challenges with studying concentrated particle suspensions is that the refractive index, *n*, mismatch between the water, n=1.333, and the material of which the colloids are formed impedes experimental optical techniques due to multiple light scattering. To overcome this challenge, we synthesize monodispersed particles from poly(2,2,2-trifluoroethyl methacrylate) (ptFEMA) which has a relatively low refractive index of n=1.42 which is suitable for refractive index matching. By forming particles with diameters between 0.5 μm and 3 μm, they are large enough to be easily visualized by optical microscopy and also small enough to undergo thermal fluctuations; here, we synthesize 1.00 μm diameter particles. Additionally, these particles are co-polymerized with 2-(2-bromoisobutyryloxy) ethyl methacrylate (BIEA) which acts as a monomer during particle synthesis and as an initiator for atom transfer radical polymer (ATRP) [13]. Due to its two sided functionality, this molecule is called an *inimer* [14]. Varying the co-polymerization volume percentage from 0.1% to 3.0% of *inimer* during particle synthesis enables tuning of the grafting density on the particle surface. Additionally, ATRP allows for precise control over the length of these grafted polymers [15]; resulting in the independent ability to tune both the length and density of polymer present on the particle surface as depicted in Figure 1.

**Figure 2:**
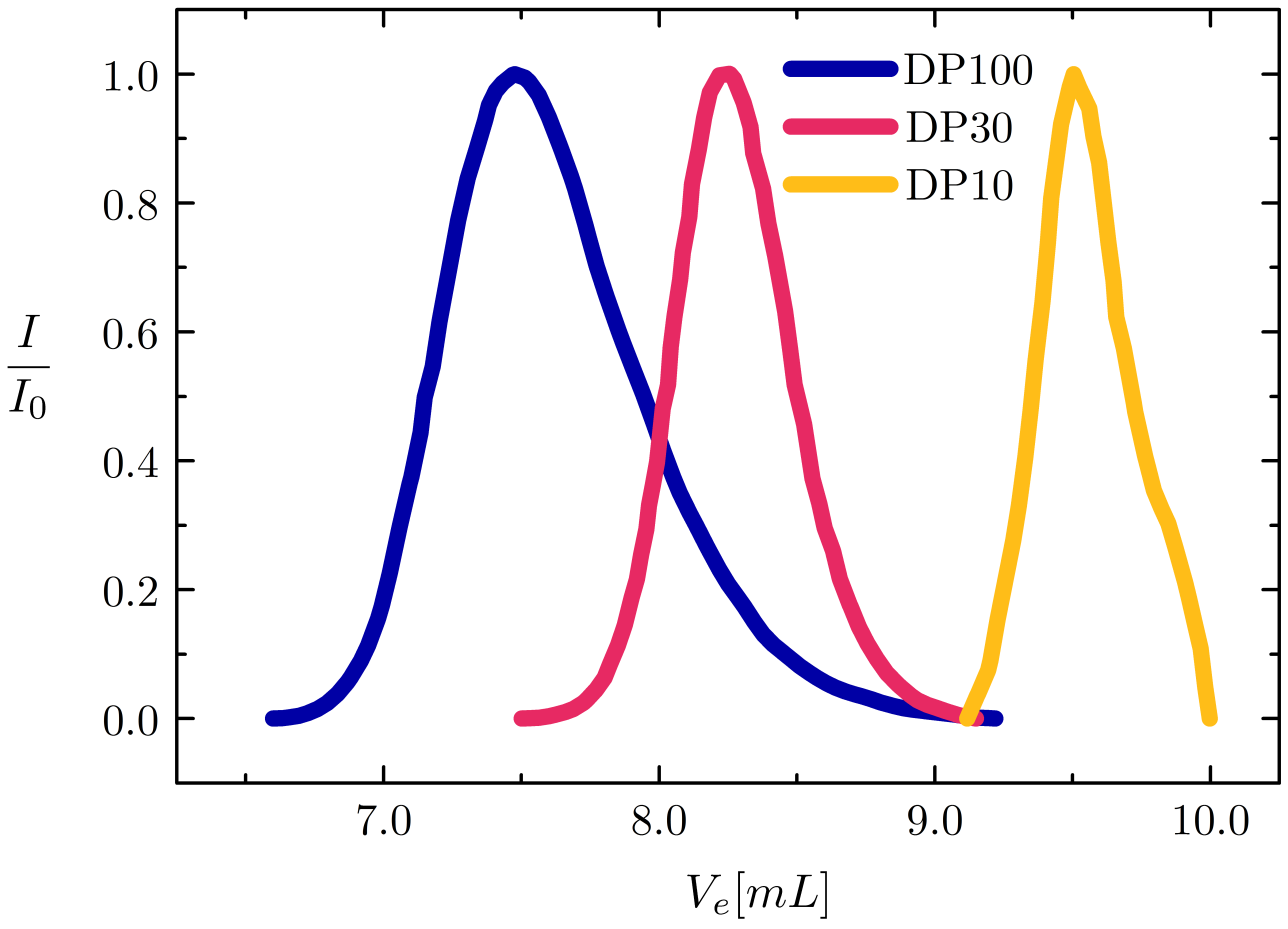
Gel permeation chromatography (GPC) elution profiles for polymers with different degrees of polymerization; with the elution volume of the polymers being inversely proportional to their respective degree of polymerisation. DP = 10, Mn = 2.9 × 10^2^ g/mol, Mw = 3.1 × 10^2^ g/mol, PDI = 1.10; DP = 30, Mn = 3.1 × 10^3^ g/mol, Mw = 3.6 × 10^3^ g/mol, PDI = 1.2; DP = 100, Mn = 9.3 × 10^3^ g/mol, Mw = 1.5 × 10^4^ g/mol, PDI = 1.6.

During a typical ATRP reaction, the degree of polymerization is controlled by the molar ratio of the initiator to monomer. However, the precise molar value of surface available *inimer* molecules is difficult to determine. This leaves choosing the appropriate amount of monomer to establish a desired ratio challenging. To nevertheless control the length of the grafted polymers, we add a conventional ATRP initiator with identical ATRP initiation rate to the grafting reaction. This yields a free linear polymer with the same degree of polymerization (DP) as the polymers which are simultaneously grown from the surface [16]. Gel permeation chromatography (GPC) analysis of the linear polymer results in a clear dependence in the chain length for the desired DP, while still retaining a fairly monodisperse distribution as seen in Figure 2.

To obtain a temperature triggerable interaction, a pNIPAM surface modification is insufficient; electrostatic repulsion between particles must also be tuned. A controlled concentration of salt, 30 mM NaCl, is added to screen electrostatic repulsions to approximately the length scale of the shortest surface polymers; the calculated Debye screening length is $\kappa^{-1}=1.7$ nm. It must be noted that at higher [NaCl], the LCST of pNIPAM decreases below room temperature [12] and additionally electrostatic repulsion is insufficient to prevent aggregation by van der Waals forces between particles; the precise salt concentration is crucial to obtain a temperature sensitive interaction potential via the pNIPAM grafted surfaces.

**Figure 3:**
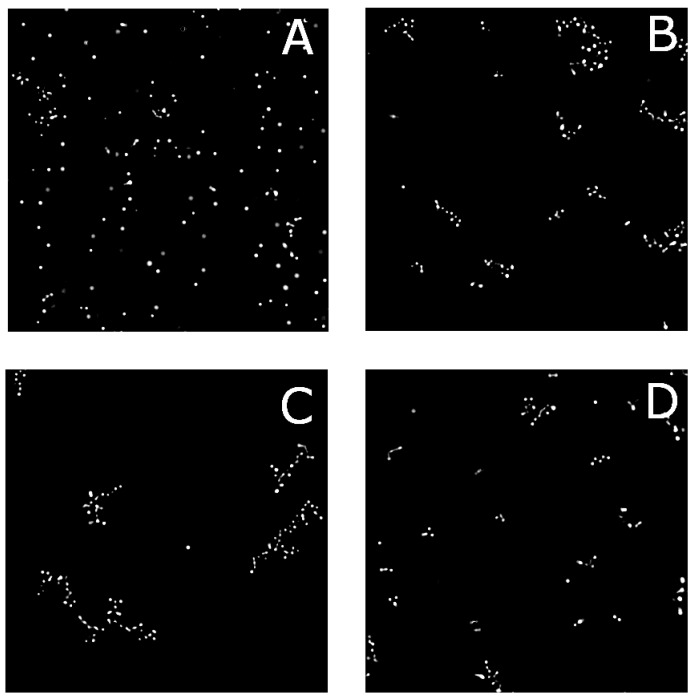
Optical microscopy images for different grafting densities at 32 °C in 30 mM NaCl for DP = 100. (**A**) 0.1%; (**B**) 0.3%; (**C**) 1.0%; and (**D**) 3.0%.

**Figure 4:**
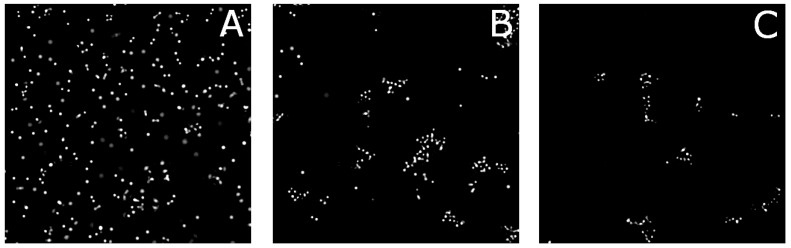
Optical microscopy images for different chain lengths at 32 °C in 10 mM NaCl for a grafting density of 3.0%. (**A**) DP = 10; (**B**) DP = 30; and (**C**) DP = 100.

To study the structure and dynamics of aggregated surface modified particles, we employ bright-field microscopy. A two-dimensional array of colloidal particles is formed by simply letting the relatively dense ptFEMA colloids sediment onto the capillary wall. To prevent particles adhering to the capillary walls, the capillaries are coated with a polyeletrolyte multilayer which has been shown to eliminate wall interactions for pNIPAM layers [17,18]. Once sedimented, the sample is heated to a temperature slightly below the LCST of pNIPAM in pure water, the particles begin to form two-dimensional aggregates as seen in Figure 3. For the lowest grafting density, only a few aggregates are found at this temperature and volume fraction, ϕ, while at higher grafting density, large extended aggregates are visible. Correspondingly, for particles with a constant grafting density but differing chain length, the effects are similar: At the short chain lengths, the degree of aggregation is limited while at longer chain lengths, very few individual particles exist as seen in Figure 4. Aggregates of particles with the highest grafting density seem to be smaller than aggregates composed of particles with lower grafting densities. This may be due to particles with lower grafting densities rearranging more easily. Within each aggregate, the magnitude of the thermal fluctuations between particles appears to be directly related to the chain length and grafting density of the surface polymer.

**Figure 5:**
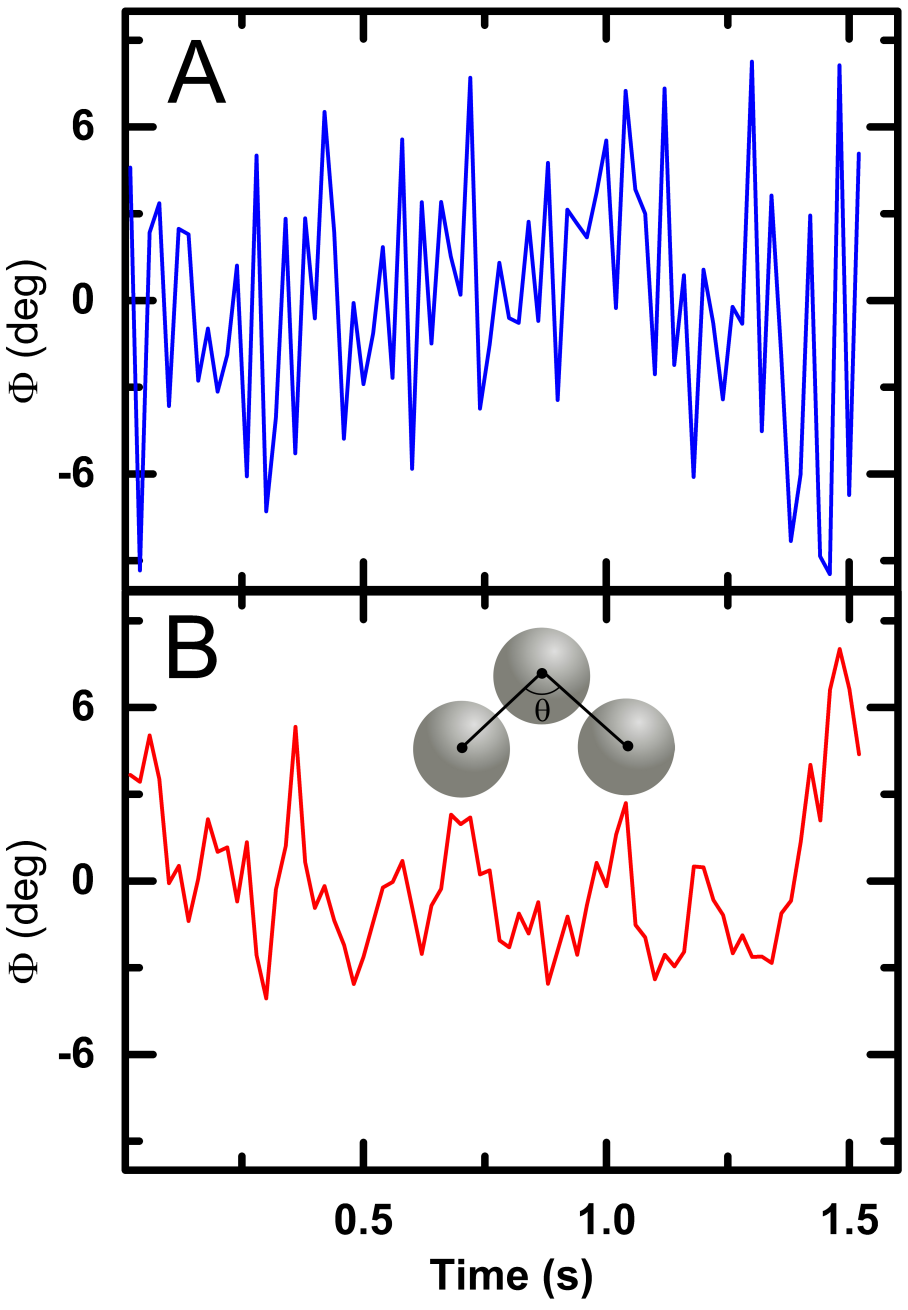
Bond angle fluctuations for samples of DP = 100 with 0.3% (**A**) and 3% (**B**) grafting density. The variance of the fluctuations are 6.7 ${\left(\deg\right)}^{2}$ and 19.2 ${\left(\deg\right)}^{2}$ respectively. Inset; schematic representation of bond angle calculation between neighboring particles.

By measuring the angle between neighboring particles over time, we are able to directly quantify the amplitude of the bond angle fluctuations as a proxy for the friction coefficient. Centers of neighboring particle are first located and tracked over time; after which the angle, θ(t), is calculated as seen in Figure 5 inset. The fluctuations about the mean angle, θ(t)=θ(t)−〈θ(t)〉, are shown for two grafting densities in Figure 5 (see Supplementary Movies). At lower grafting density, angular fluctuations are large. Conversely, at a higher grafting density, the angular fluctuations are minimized. A smaller amplitude in θ corresponds to more hindrances in thermally activated motion between particles occurring which points at a higher friction between the particle surfaces [18]. Polymer brushes, with their high grafting densities, have repeatedly been found to be low friction interfaces, seemingly contradictory to the above observations [19,20,21]. However, temperature sensitive polymer brushes tethered to a substrate have been shown to switch from low to high friction above the LCST of pNIPAM which supports the different amplitudes of θ seen in Figure 5 [22,23]. Therefore, increasing grafting density also increases the friction between particles; the consequences of this increased friction may be profound. We hypothesize that colloidal gels with lower friction coefficients and therefore more flexible bonds are capable of relaxing applied stresses and would result in a lower elastic modulus.

**Figure 6:**
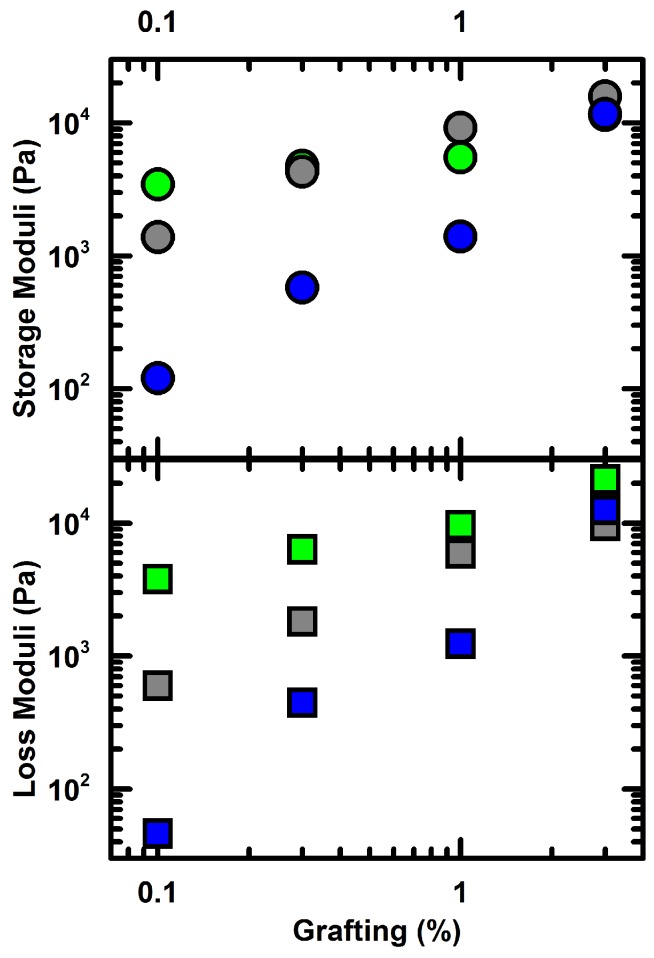
Storage and loss moduli after heating dispersions at ϕ=0.28 with 30 mM NaCl to 45 °C with green for DP = 10, gray for DP = 30, blue for DP = 100. All moduli are measured at 1 Hz and γ<0.03.

To directly investigate whether more flexible bonds lead to a lower elastic modulus, we use bulk rheology. At a higher volume fraction, ϕ=0.28±0.02, the particle dispersions form elastic 3D colloidal gels upon heating above the LCST. We compare the mechanical behavior of colloidal gels with differing grafting densities and chain length of the surface pNIPAM polymer. Though the precise volume fraction of the dispersion is not known, the resulting differences of linear mechanical response in these gels are larger than the variance caused by the uncertainty in ϕ as seen in Figure 6. The elastic modulus of colloidal gels has been shown to scale as, $G^{\prime }=\left(\kappa_{0}/a\right){\left(\phi -\phi_{c}\right)}^{p}$ where $\kappa_{0}$ is the two-particle spring constant, *a* is the particle size, *p* is a scaling exponent which depends on the nature of the network deformation, and $\phi_{c}$ is the critical volume fraction which is typically $\phi_{c}\leq 0.08$ [7,24]. Here, $\phi =0.28>>\phi_{c}$, therefore, the uncertainty in ϕ cannot account for the large variation in the elastic moduli seen in Figure 6; it must arise from changes in $\kappa_{0}$. For the highest grafting densities, the elastic and viscous responses of the gels converge for all chain lengths. By contrast, at lower grafting densities the gels are significantly weaker by nearly two orders of magnitude for the longest chain length; this drop in elasticity corresponds well with the larger magnitude in θ observed microscopically as seen in Figure 5.

Colloidal networks resist mechanical deformation by stretching interparticle bonds and bending particle strands composed of multiple particles. These bending modes result in angular changes between individual particles, Δθ, and have been shown to contribute significantly to the elastic response of colloidal networks [7]. Therefore, hindering these bending modes can directly increase the elastic response which is seen in Figure 6. How precisely the microscopic changes, grafting density and chain length, manifest as differences in the macroscopic rheology including yielding is beyond the scope of this work and has been the subject of extensive simulation studies [25,26,27].

**Figure 7:**
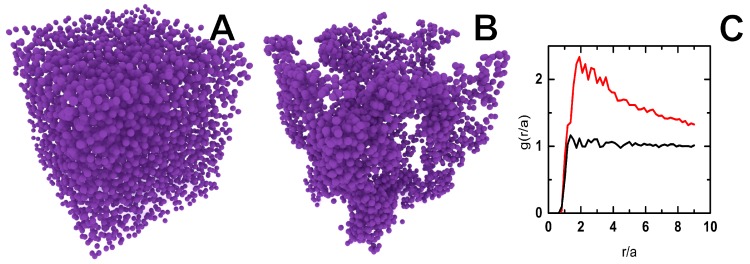
Computer-reconstructed visualizations of a sample with particle coordinates obtained from three-dimensional confocal microscopy data. The field of view is 67 μm × 67 μm × 75 μm. (**A**) a liquid dispersion of particles, ϕ≈0.15, at 25 °C in 50 wt % Sucrose with 10 mM NaCl; (**B**) a colloidal gel of the same dispersion at 50 °C; (**C**) calculated radial distribution functions normalized for particle size, *a*, for gel (red, **A**) and liquid dispersion (black, **B**).

Finally, these model ptFEMA particles may be fluorescently labeled and dispersed in a refractive index matching solution of 50 wt % sucrose with 10 mM NaCl. By refractive index matching the particles to the suspending solution, light scattering is minimized and by combining fluorescent labeling, 3D confocal microscopy images may be captured. As the thermo-responsive nature of the polymer brush is retained in the sucrose solution, the dispersion may still be heated from a colloidal liquid into a colloidal gel while being imaged deep into the sample, ≈75 μm as shown in Figure 7. From the individual particle locations, the radial distribution function, g(r/a), was calculated and shown in Figure 7C; the g(r/a) clearly show a transition from a liquid dispersion of particles to a colloidal gel by heating. This ability to dynamically induce gelation by heating this particle dispersion with its controlled pNIPAM surface polymer is similar to previous work where the authors quantified the kinetics and structure of pNIPAM grafted nanoparticles [28]. In these dynamic light scattering studies, only the fractal dimension was determined as individual particle kinetics are not available. From this work, a detailed kinetic aggregation framework was proposed to connect the local particle-level dynamics to the macroscopic rheology, effectively describing many experimental rheology results on colloidal gels [29]. The model system proposed here will allow for a detailed study of this kinetics framework to different gelation processes as well as allowing direct observation of microscopic sliding dynamics between particles after gelation in three dimensions using confocal microscopy.

## 3. Conclusions

We have developed a thermally responsive colloidal system with controlled grafting density and chain length of a pNIPAM polymer on the particle surface. Upon heating, such dispersions form a colloidal gel. Both the microscopic bond angle fluctuations and macroscopic elastic moduli exhibit a clear dependence on both grafting density and chain length. The unique combination of complete transparency, tunable particle surface properties and temperature-triggerable interactions paves the way to the study of gelation kinetics in three-dimensions with high resolution.

## 4. Materials and Methods

All materials were purchased from TCI Europe (Zwijndrecht, Belgium) and used as received unless otherwise noted. *N*-Isopropyl acrylamide (NIPAM) monomer was recrystallized from *n*-hexane prior to use. Additionally, the inimer monomer, 2-(2-bromoisobutyryloxy) ethyl methacrylate (BIEA), was synthesized as previously reported [13,14].

### 4.1. Particle Synthesis

We synthesized poly(2,2,2-trifluoroethyl methacrylate) (ptFEMA) colloidal particles co-polymerized with 2-(2-bromoisobutyryloxy) ethyl methacrylate using free radical dispersion polymerization [13]. To a 500 mL round bottom flask is added 30 mL water, 270 mL methanol, 25 mL 2,2,2-trifluoroethyl methacrylate, 250 mg 2,2-azobis(2-methylpropionitrile), 250 mg 3-sulfopropyl methacrylate potassium salt (Sigma-Aldrich, St. Louis, MI, USA) and 25 μL of BIEA (0.1 vol % to monomer). The flask is placed under reflux conditions in a silicone oil bath preheated to 80 °C and allowed to polymerize for 4 h. The resulting particles have a hydrodynamic diameter, a=1.00 μm with a polydispersity index, PDI=(σ(a)/〈a〉)=0.01 as determined by Dynamic Light Scattering (DLS). The reaction is repeated with 75 μL, 250 μL and 750 μL of BIEA to arrive at 0.3 vol %, 1.0 vol % and 3.0 vol % of *inimer* respective to monomer, with no measurable change to particle diameter or polydispersity.

### 4.2. Surface Initiated ATRP

Particle dispersions were washed three times by centrifugation at 250×*g* into a 1 wt % solution of L23 surfactant (Sigma-Aldrich) to a final volume of 200 mL. To graft polymers from the particle surface, 50 g dimethylformamide, 2 g NIPAM (1.7 × 10^−2^ moles), 0.47 mL tris[2-(dimethylamino)ethyl]amine (1.7 × 10^−3^ moles), 0.253 mL ethyl α-bromoisobutyrate (1.7 × 10^−3^ moles) were added to a 250 mL round bottom flask. The solution was bubbled with nitrogen for 15 min, after which 0.168 g of Cu(I)Cl (1.7 × 10^−3^ moles) was added to initiate the polymerization. The above procedure yielded a DP = 10 as shown in Figure 2. For DP = 30, 50 mL particle dispersion, 50 g dimethylformamide, 2 g NIPAM (1.7 × 10^−2^ moles), 0.156 mL tris[2-(dimethylamino)ethyl]amine (0.56 × 10^−3^ moles), 0.084 mL ethyl α-bromoisobutyrate (0.56 × 10^−3^ moles) were added to a 250 mL round bottom flask, bubbled, and initiated with 0.056 g of Cu(I)Cl (0.56 × 10^−3^ moles). For DP = 100, 50 mL particle dispersion, 50 g dimethylformamide, 2 g NIPAM (1.7 × 10^−2^ moles), 0.047 mL tris[2-(dimethylamino)ethyl]amine (1.7 × 10^−4^ moles), 0.025 mL ethyl α-bromoisobutyrate (1.7 × 10^−4^ moles) were added to a 250 mL round bottom flask and initiated with 0.017 g of Cu(I)Cl (1.7 × 10^−4^ moles). These procedures were repeated for each BIEA volume ratio, 0.1%, 0.3 vol %, 1.0 vol % and 3.0 vol %, to yield a total of 12 different particle dispersions each with a unique grafting density and chain length. After surface modification, the dispersions were centrifuged and the supernatant collected and purified before GPC measurements. The sedimented particles were redispersed in 20 mL of demineralized water and each particle dispersion was dialyzed for 10 days again in deionized water to remove Cu(I)Cl and the surfactant L23. The hydrodynamic diameters of the particles after surface modification have been characterized by DLS using a second-order cumulants fit to the correlation functions. The results show an increasing trend only for the particles with the highest surface grafting density, 3%, from a=1020±68 nm for the bare particles to DP = 10, a=996±63 nm; DP = 30, a=1044±31 nm; and DP = 100, a=1112±40 nm.

The supernatant was heated to 80 °C overnight to remove water and then precipitated in diethyl ether, dissolved in chloroform, and precipitated again, a total of three times. The precipitate was dried and dissolved in water and mixed bed resins (AG501-X8, Bio-Rad, Hercules, CA, USA) were added to remove copper salts. The resins were filtered away and the now clean pNIPAM polymer was freeze dried. GPC measurements were performed on 5 mg/mL samples in a solution of tetrahydrofuran with 5 vol % triethylamine at a flow rate of 1 mL/min at 35 °C on an Agilent Technologies 1200, PLgel 5 μm Mixed-D column [30]. The column was calibrated prior to use with linear polystyrene dissolved in the above solvent.

### 4.3. Fluorescent Labeling

A single dispersion, 1 vol % BIEA with DP = 100, was fluorescently labeled. A miniemulsion was prepared by tip sonication, containing 0.2 mL toluene, 5 mg boron-dipyrromethene 543 dye (Excition, Inc., West Chester, OH, USA), and 4 mL 1 wt % solution of L23 surfactant. To this miniemulsion, 1.5 mL of particle dispersion at ϕ=0.30 was added. This dispersion was mixed for 3 days to allow the particles to swell and take up the dye. Subsequently, dry nitrogen was blown over the top of the dispersion to remove toluene and kinetically trap the dye inside the particles. This fluorescently labeled dispersion was dialyzed against deionized water to remove L23. Sucrose was then added as a powder and dissolved to a final concentration of 50 wt % which resulted in a refractive index matched dispersion.

### 4.4. Microscopy

Bright field and confocal microscopy experiments were performed in capillaries of 40 mm × 4 mm × 0.2 mm inner dimensions coated with polyelectrolyte multilayers. Capillaries were first plasma treated, then submerged into a 1 M NaCl solution with 1 wt % poly(diallydimethyl ammonium) chloride (Mw ≈ 5 × 10^5^ g/mol, Sigma-Aldirch), then washed extensively with deionized water, then submerged in a 1 M NaCl solution with 1 wt % poly(styrene sulfonate) (Mw ≈ 2 × 10^5^ g/mol, Sigma-Aldrich) and finally washed extensively with deionized water. This layer-by-layer treatment was repeated three times for a total of six layers. A dilute suspension of each particle dispersion, ϕ=0.001, was prepared by diluting with either a 10 mM or 30 mM NaCl solution, loaded into a coated capillary, allowed to sediment over 1 h and finally heated to the desired temperature using a home-built objective and capillary heater. Samples were allowed to equilibrate for 10 min at each temperature before imaging. Images were then captured using a Nikon microscopy (Nikon Instruments, Amsterdam, The Netherlands) with a 60× water immersion objective at 50 fps using a Fastec HiSpec1 camera (Fastec Imaging Corporation, San Diego, CA, USA). Confocal microscopy 3D images were captured using a Zeiss LSM5 Pascal (Carl Zeiss AG, Oberkochen, Germany) with 488 nm excitation and 100× oil immersion objective. The refractive index matched dispersion in 50 wt % sucrose with 10 mM NaCl was first imaged at room temperature then quickly heated to 50 °C. Particle centers were located using standard locating software [31] using Matlab.

### 4.5. Rheology

For rheology measurements, each dialyzed dispersion was allowed to sediment over several days and the supernatant removed until the dispersion obtained a high volume fraction, ϕ>0.30. Each dispersion’s volume fraction was measured by drying a known mass of dispersion, ≈1.00 g, in an 80 °C oven overnight; this method exhibited repeatability within 6% of the mean. To this measured dispersion, a small volume of water and 2.0 M NaCl was added to obtain ϕ=0.28 in 100 mM NaCl for each dispersion which was measured using an Anton Paar MCR501 rheometer (Anton Paar, Graz, Austria) with a 50 mm diameter cone-plate geometry. A solution of tetradecane was added around the geometry to minimize evaporation. The dispersion was heated to 45 °C in 10 min and allowed to gel further over 1 h, then measured at 1 Hz with an applied strain from γ=0.001 to γ=1.00 and an average value taken within the linear regime, typically γ<0.03.

## Supplementary Materials

The following are available online at www.mdpi.com/2310-2861/3/2/21/s1.

## Abbreviations

The following abbreviations are used in this manuscript:

pNIPAM	poly(*N*-isopropyl acrylamide)
ptFEMA	poly(2,2,2-trifluoroethyl methacrylate)
BIEA	2-(2-bromoisobutyryloxy) ethyl methacrylate
ATRP	Atom Transfer Radical Polymerization
GPC	Gel Permeation Chromatography
DP	Degree of Polymerisation
